# Long story short

**DOI:** 10.1093/ehjcr/ytae351

**Published:** 2024-07-18

**Authors:** Murad Almasri, Nirbhay Parashar, Jeffrey Orcutt

**Affiliations:** Pediatric Cardiology Department, Arkansas Children’s Hospital, 1 Children’s Way, Little Rock, AR 72202, USA; Pediatric Cardiology Department, Arkansas Children’s Hospital, 1 Children’s Way, Little Rock, AR 72202, USA; Pediatric Cardiology Department, Arkansas Children’s Hospital, 1 Children’s Way, Little Rock, AR 72202, USA

## Clinical vignette

A 13-year-old lady is followed periodically in the paediatric cardiology clinic. The patient suffered from a cardiac arrest when she was 2 years old and had a dual-chamber implantable cardioverter-defibrillator (ICD) implanted then. Her echocardiogram showed a normal heart structure and function. She had no further cardiac arrest episodes following her implantation and is seen regularly in the clinic with electrocardiograms (ECGs), Holters, and device checks. She is compliant with her Sotalol.

1. What is the diagnosis based on the clinical picture and ECG findings?
  - Complete heart block
  - Hypokalaemia
  - Long QT syndrome
  - Short QT syndrome
  - Brugada syndrome

The correct answer is D.

**Figure 1:**
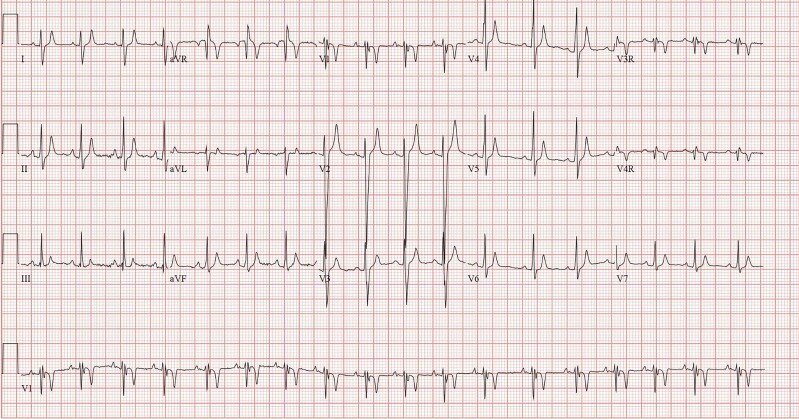
The patient's ECG showing normal sinus rhythm with a QT interval of 240 ms and a QTc of 245 ms.

Short QT syndrome (SQTS) is a rare, inherited channelopathy which is a known cause of sudden cardiac death due to increased risk of atrial and ventricular arrhythmias.^1^ Diagnosis is established based on ECG findings of an abnormally short QT interval (*Figure 1*). Short QT syndrome is usually defined as QTc < 330 ms or QT interval <360 ms and one or more of the following: history of cardiac arrest or syncope, family history of sudden cardiac death at age 40, or younger or a family history of SQTS.^2^ This patient has a QT interval of 240 ms and QTc 245 ms and has a history of cardiac arrest.

2. Which of the following is a known strong predictor for risk of recurrent ventricular arrhythmias over the course of time for a patient with this diagnosis?
  - Positive genotype
  - History of survived cardiac arrest at initial presentation
  - History of anorexia
  - Family history of arrhythmia
  - Associated structural heart disease

The correct answer is B.

Short QT syndrome diagnosing criteria by Gollob *et al.*^2^ are composed of four components: ECG, clinical history, family history, and genotype. At least one point should be from ECG criteria. Four points or more indicate high probability, 3 points intermediate, and 2 points or fewer low probability of diagnosis of SQTS. Risk stratification remains a challenge. Gollob or modified Gollob score does not correlate with adverse cardiac events. However, history of survived cardiac arrest at initial presentation is a strong predictor of recurrent ventricular arrhythmias as two-thirds of them had a recurrence at follow-up. This strongly supports ICD implantation in cardiac arrest survivors.

3. What is the most common complication for an ICD use in paediatric patients with this diagnosis?
  - Non-capture
  - Inappropriate ICD shocks
  - AICD pocket infection
  - Lead fracture
  - Lead dislodgement

The correct answer is B.

Implantable cardioverter-defibrillator is recommended in symptomatic SQTS patients who are either survivors of sudden cardiac arrest and/or have documented spontaneous sustained ventricular tachyarrhythmias with or without syncope (Class I recommendation).^3^ Implantable cardioverter-defibrillator use for primary prevention should be limited, especially in young patients. There is an unusually high incidence of inappropriate ICD shocks in patients with SQTS.^4^ This may be due to oversensing of short-coupled and prominent T-waves, resulting in T-wave oversensing. There is also a high prevalence of ventricular lead fracture in paediatric patients, with most resulting in inappropriate ICD shocks.

## Data Availability

No new data were generated or analysed in support of this research.

## Notes

**Consent:** The patient’s guardian consented to the publication of this case.

**Funding:** None declared.
